# One New Phenolic Compound from *Castanea mollissima* Shells and its Suppression of HepatomaCell Proliferation and Inflammation by Inhibiting NF-κB Pathway

**DOI:** 10.3390/ijms20030466

**Published:** 2019-01-22

**Authors:** Wu Fei, Yao Xuan, Xu Jian, Wu Yue, Yang Yuejun, Jin Yu, Xie Huifang, Liu Yuancai, Yang Yifu, Zheng Xiangwei

**Affiliations:** 1 Shanghai University of Traditional Chinese Medicine Engineering Research Center of Modern Preparation Technology of TCM, Ministry of Education, Shanghai, 200000, China; wufei_shutcm@126.com; 2 Shanghai University of Traditional Chinese Medicine, Shanghai, 200000, China; shane.yao@163.com; 3 Jing Brand Research Institute, Jing Brand Co., Ltd., Daye, 435100, China; xujian@jingpai.com (X.J.); proyue1122@gmail.com(W.Y.); yyj@jingpai.com (Y.J.); 4 Engineering Research Center of Pharmaceutical Process Chemistry, Ministry of Education, School of Pharmacy, East China University of Science and Technology, Shanghai, 200000, China; Jiny@ecust.edu.cn; 5 Biotechnology Research & Innovation Department, Shanghai Huangdian Co., Ltd., Shanghai, 200000, China; hf_xie@sina.com

**Keywords:** *Castanea mollissima* shells, phenols, anti-inflammatory activities, anticancer activities, hepatoma cells, TLR4–NF-κB pathway

## Abstract

Shells of *Castanea* *mollissima* (CMS), an agricultural remain and often considered waste from chestnut processing industry, have been proven a resource for traditional Chinese medicine. One new phenol, named castanolB(1), andsix known phenolic compounds (2–7) were isolated froma water-soluble extract of CMS. Their chemical structures were determined using preparative HPLC and various spectral analyses, and then were compared to literatures, which indicated the first identification of the seven compounds from *C. mollissima*. The physicochemical property of compound (2) was also reported for the first time. After antiproliferative screening of compounds (1–7) on LPS-induced SMMC-7721 and HepG2 hepatoma cells, castanolB (1) showed the best suppression. CastanolB(1) also significantly induced cell apoptosis. Furthermore, castanolB (1) decreasedsecretion of TNF-α and IL-6. Mechanistically, TLR4–NF-κB pathway was inhibited bycastanolB (1) with downregulation of TLR4, IKKβ, and NF-κB p65. This study presents a new phenol and shows its profiles of anticancer and anti-inflammation via inhibiting the TLR4–NF-κB pathway.

## 1. Introduction

Chinese chestnut (*Castanea mollissima*) is one of the highest-yielding dried fruits in the world [1]. According to 2016 statistical data from FAOSTAT (Food and Agriculture Organization of the United Nations), the world chestnut production reaches 2.3 million tons, of which China accounts for about 1.9 million tons [2]. As the exocarp, the shells of *C.mollissima* (CMS) generate a huge amount of waste, whichhas little further use. CMS can be used as fuel or processed into dyes and herbs; however, the utilization is extremely low, resulting in resource waste and environmental pollution, it is therefore necessary to dig out the value of CMS. In traditional Chinese medicine (TCM), CMS is a kind of herbs for treating various diseases, such as regurgitation, epistaxis, hemafecia, pertussis, and so on [1]. In this study, we hope to identify the chemical compositionand the bioactivities of CMS extract.

Several researchers have explored the biological activities of CMS extract and the chemical composition of its fat-soluble parts. You et al. [3] assessed the antimicrobial activity and DPPH scavenge capacity of CMS extract. Zhang et al. [4] found that CMS extract triggered necrosis and arrested the cell cycle of HepG2 cells. In addition to the bioactivity researches on CMS extract, 16 compoundswere isolated and identified the structures from the fat-soluble extract of CMS [1]. However, it has been rarely reported the chemical composition of water-soluble extract of CMS, as well as the active ingredients structures, because CMS contains a lot of water-soluble tannins and melanin impurities which cause difficulties on compoundisolation and purification. In this study, we firstly aimed to isolate and purify the water-soluble chemical components of CMS, and to identify the isolates structures by preparative HPLC. Then, all isolates were focused on the anti-hepatoma activity screening because Zhang et al. [4] confirmed the proapoptotic effect of CMS on hepatoma cells. Furthermore, a new compound, castanolB (1), was evaluated for its combined mechanism of anti-inflammation and anticancer.

## 2. Results

### 2.1. Chemistry

**Figure 1:**
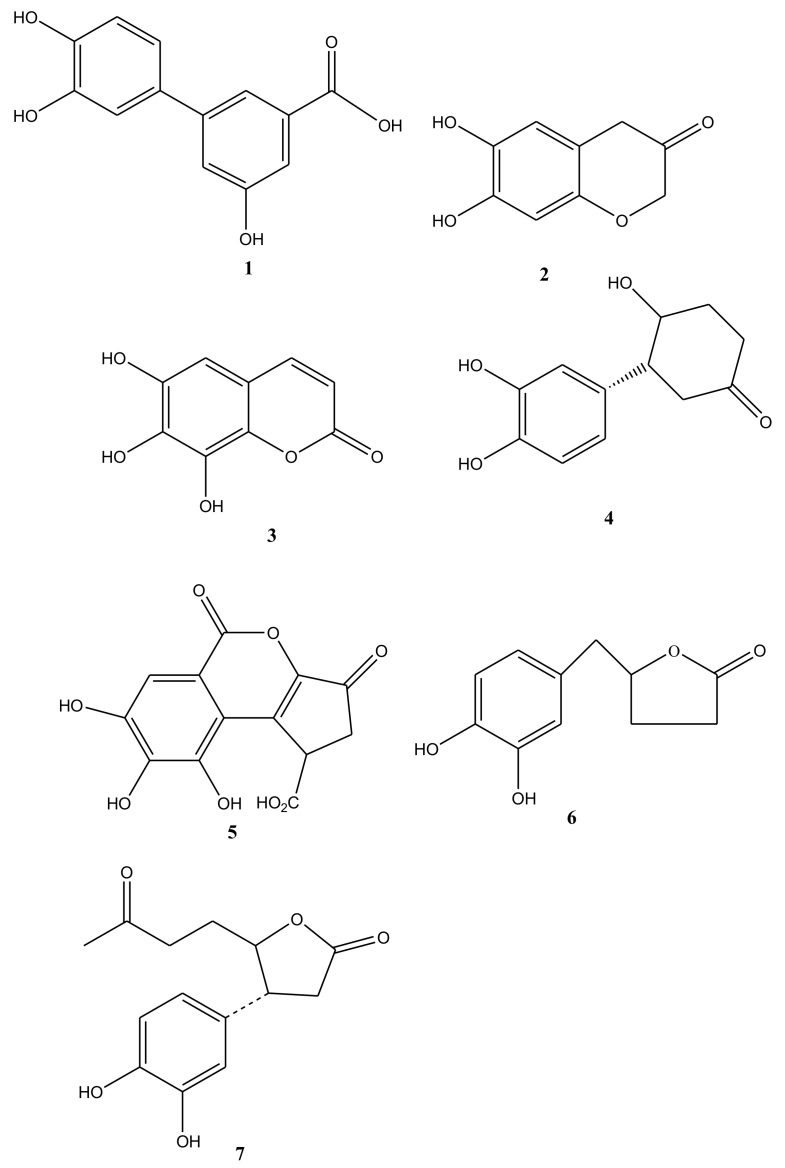
Structures of compounds (**1**)–(**7**) from water-soluble extracts of *Castanea mollissima* shells.

The methanol extract of CMS was subjected to preparative HPLC to yield one new compound castanolB (1), together with six known compounds. Compound 2 was identified as 7,8-dihydroxy-2H-chromen-3-one (2) (CAS: 56022-24-3), which physicochemical profiles was firstly reported. Then the structures of the other five compounds, after their physicochemical data were compared to reported literatures, were elucidatedas 7,8,9-trihydroxy-chromen-2-one(3) [5], 3-(3,4-dihydroxyphenyl)-4-hydroxycyclohexanone (4) [6], brevifolin carboxylic acid (5) [7], (3R,4R)-3-(3,4-dihydroxyphenyl)-4-hydroxycyclohexanone(6) [8], and (4R)-5-(1-(3,4-dihydrophenyl)-3-oxobutyl)-dihydrofuran-2(3H)-one(7) [6]. Of them, compounds (2)–(7) were identified the existence in *C. mollissima* for the first time (Figure 1).

**Figure 2:**
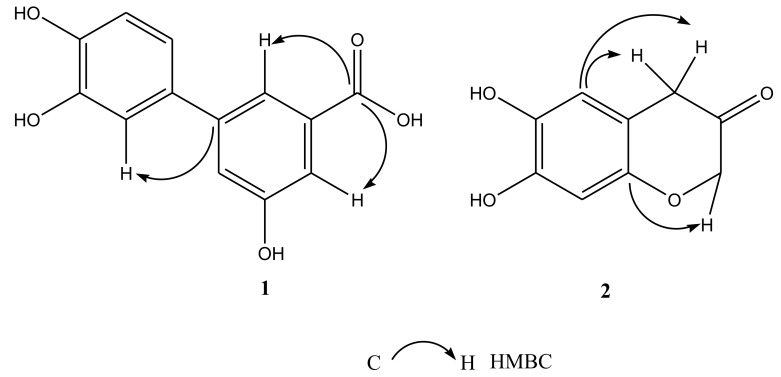
Key HMBC correlations of compounds (**1**)–(**2**) (C–H).

**Figure 3:**
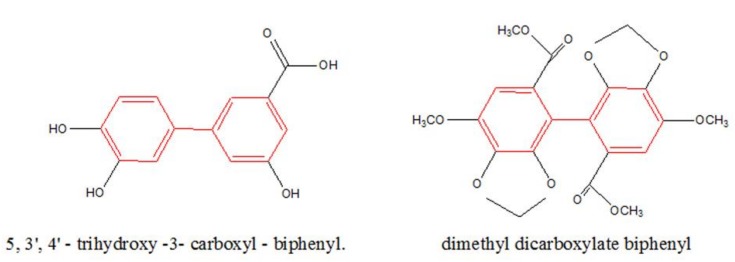
The biphenyl skeleton in compound (**1**) and dimethyl dicarboxylate biphenyl (DDB).

Compound **1**, a pale yellow powder, was analyzed the molecular formula by high resolution-electrospray ionization-mass spectrometry (HR-ESI-MS) and was deduced to be C_13_H_10_O_5_ at m/z 245.0462 [M-H]^-^. The benzene rings, phenyl and carboxyl groups were identified by the possible characteristic absorptions showed by its UV spectrum in MeOH at λmax 210, 247, and 255 nm. Compound **1** also contained OH groups (3289 cm^−1^), C=O groups (1762 cm^−1^), and aromatic rings (1615, 1585, and 1486 cm^−1^) using the IR spectrum. According to ^1^H-NMR spectrum, compound **1** displayed the ABX-coupled system H-atoms at δ_H_ 6.83(*d*, 1H, *J* =12.0 Hz), δ_H_ 6.95 (*dd*, 1H, *J* = 12.0, 6.0 Hz), and δ_H_ 7.05 (*d*, 1H, *J* = 6.0 Hz), as well as three single aromatic H-atoms at δ7.14, 7.33, and 7.66. Analyses of ^13^C NMR and DEPT spectra data (Table 1) indicated thirteen carbons, including one carboxyl quaternary carbon at δ169.9 and twelve aromatic carbons. These observations suggested that **1** had a biphenyl skeleton and a carboxy side chain. Additionally, it showed cross peaks between δ_C_ 169.9 (C-7) /δ_H_ 7.66 (H-2), δ_H_ 7.33 (H-4), and the C-1’ at the C-1 between δ_C_ 132.2 (C-1) /δ_H_ 7.05 (H-2’) in the HMBC spectrum of 1 (Figure 2), and verified a carboxyl group linking at the C-3. The structure of **1**, as stated in these spectral data, was elucidated as 5,3’,4’-trihydroxy-3-carboxyl-biphenyl, which parent nucleus was biphenyl skeleton and was same to dimethyl dicarboxylate biphenyl (DDB) (Figure 3).

Compound 2 was also a pale yellow powder. HR-ESI-MS data at m/z 179.0467 [M-H]^-^ showed its molecular formula as C_9_H_8_O_4_. The benzene rings and ketone groups were identified by the possible characteristic absorptions showed by its UV spectrum in MeOH at λmax 227 and 255 nm, respectively. Compound 2 also contained OH groups (3320 cm^−1^), C=O groups (1754 cm^−1^), C-O groups (1170cm^−1^), and aromatic rings (1618, 1580, and 1480 cm^−1^) using the IR spectrum. The HMQCand ^1^H-NMR spectrum of **2** showed the presence of two single aromatic H-atoms at δ 7.07, 6.84, an oxymethyleneH-atoms at 4.45 (s), and a methyleneH-atoms at δ 2.75, 3.42. By analyses of the ^13^C NMR and DEPT spectra data (Table 1), it indicated nine carbons, including one ketone quaternary carbon at δ 206.7, an oxygen linked quaternary carbon at δ 73.6, a methylene carbon at δ 73.6, and six aromatic carbons. These observations suggested that 2 had a phenylpropanoid skeleton. Additionally, the oxymethylene group was linked to the benzene ring through oxygen, which was analyzed by the cross peaks between δ_C_ 148.1 (C-10)/δ_H_ 4.40 (H-2), while a methylene group was directly linked to the benzene ring, which was investigated by the cross peaks between δ_C_ 111.2(C-6) /δ_H_ 2.75, 3.42 (H-4) according to the HMBC spectrum of 2 (Figure 2). The structure of 2, as stated in these spectral datas, was elucidated as 7, 8-dihydroxy-2H-chromen-3-one.

### 2.2. Antiproliferative Effect of These Compounds

**Figure 4:**
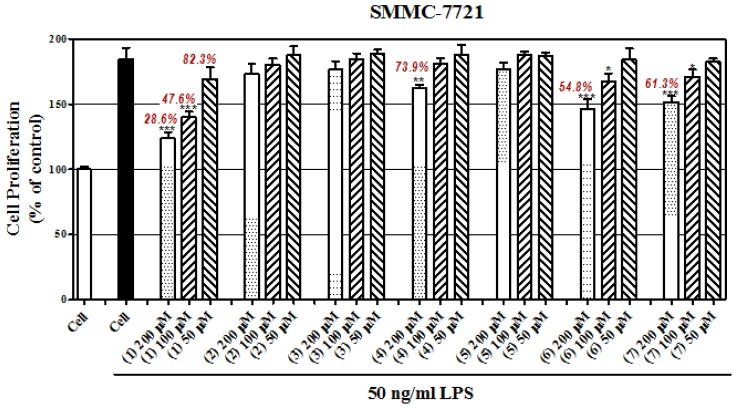

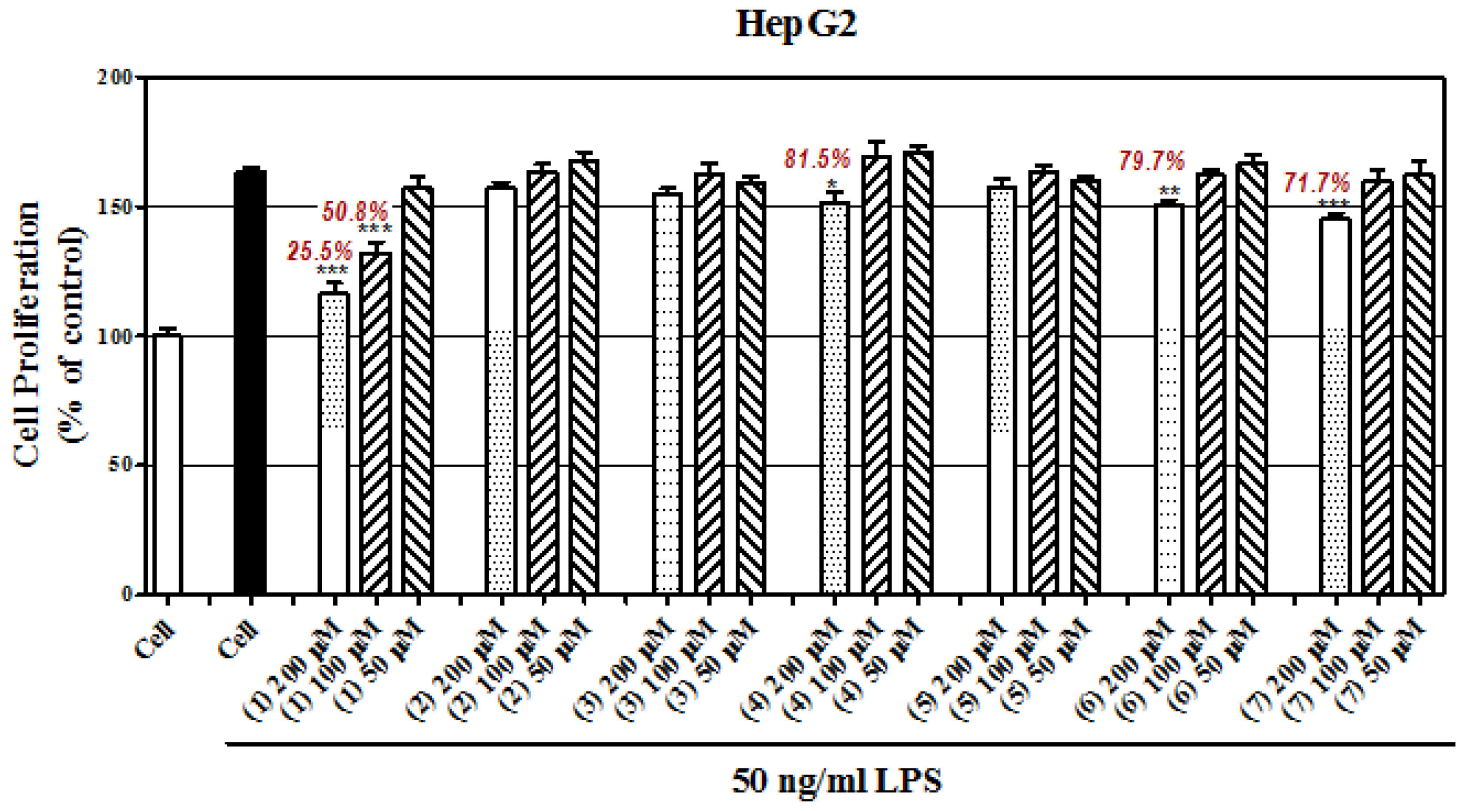
Effect of compounds on inhibition of lipopolysaccharide (LPS)-induced hepatomacell proliferation of SMMC-7721 cells (**A**) and HepG2 cells (**B**). (* denotes *p* < 0.05; ** denotes *p* < 0.01; *** denotes *p* < 0.001, vs. LPS-induced hepatoma cells).

In vitro cell models were established using lipopolysaccharide (LPS)-induced SMMC-7721 cells (human hepatoma cell line) and LPS-induced HepG2 cells (human hepatoma cell line). Briefly, the malignant cancer hallmarks, such as proliferation, can be accelerated by inflammation, which is caused by LPS. And the deteriorated process can be partly prevented by certain compounds. In this study, the concentrations of all compounds in preventing LPS-induced hepatoma cells proliferation were 200μM, 100μM,and 50μM. Then, cell proliferation was detected using a MTT assay with standard protocol. In Figure 4A,B, LPS significantly induced cell proliferation compared with those of vehicle; compared to the LPS group, castanol B (1) showed greater than 50% inhibition at both dosages of 200μM and 100μM on two cell lines. Compounds (4), (6), and (7) were found to have moderate inhibition at 200μM. Compounds (2), (3), and (5) showed faint activity compared to (1), (4), (6), and (7).

### 2.3. Castanol B(1)-Induced Cellular Apoptosis

**Figure 5:**
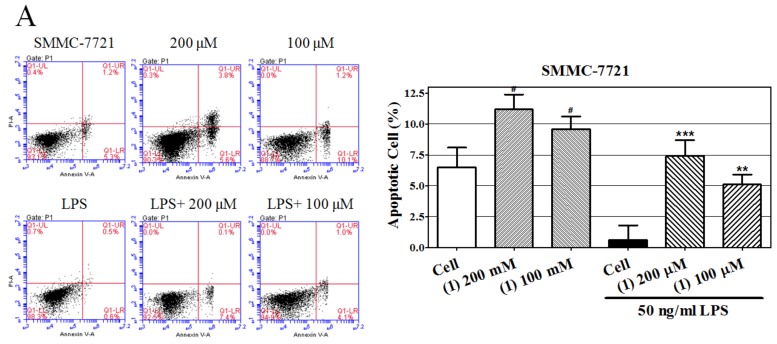

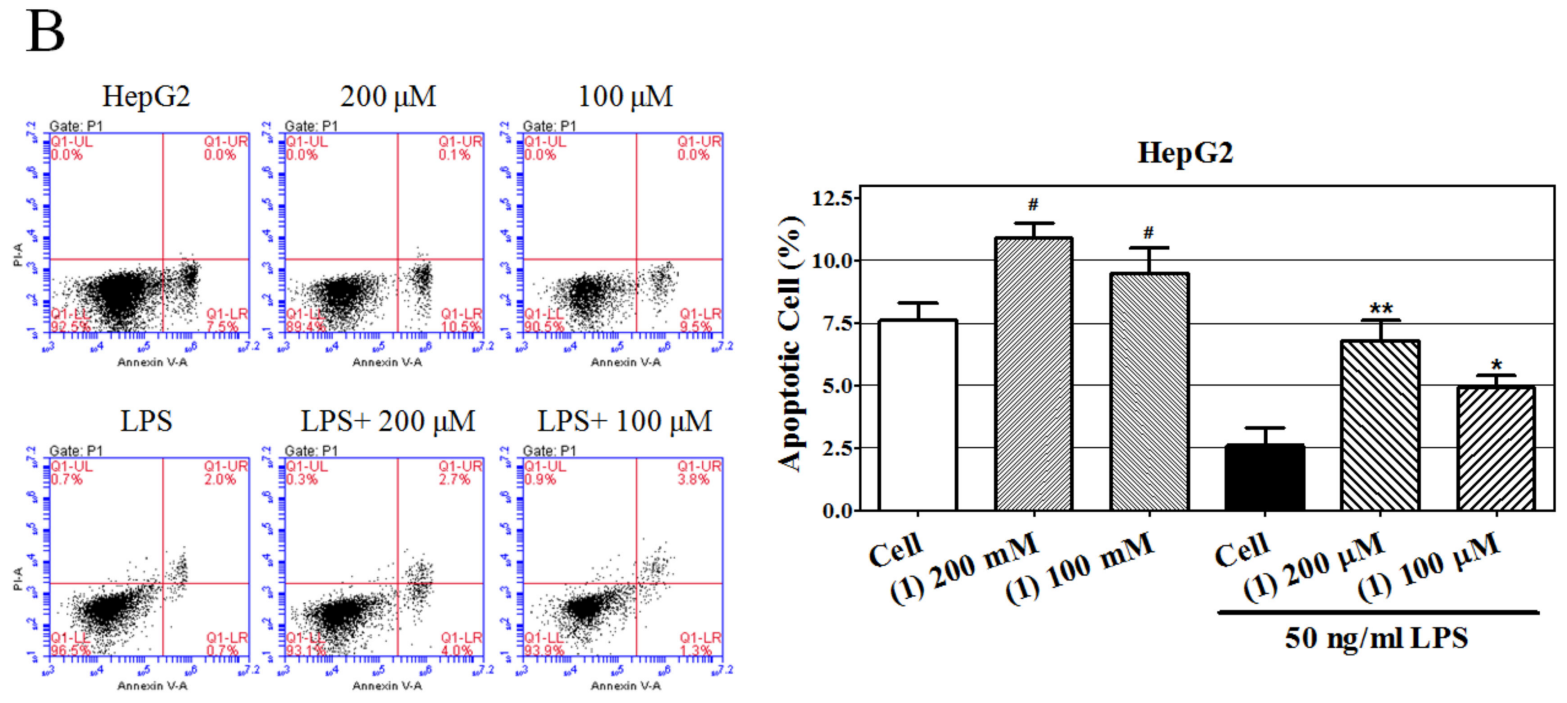
Effect of castanol B (1) on apoptosis of lipopolysaccharide(LPS)-induced hepatomaSMMC-7721 cells of SMMC-7721 cells (**A**) and HepG2 cells (**B**). (# denotes *p* < 0.05 vs. vehicle-treated hepatoma cells. * denotes *p* < 0.05; ** denotes *p* < 0.01; *** denotes *p* < 0.001, vs. LPS-induced hepatoma cells).

In Figure 5, we measured the proapoptotic effects of castanol B (1) using Annexin V/propidium iodide (PI) assay after 48 h treatment of two hepatoma cell lines with castanol B (1). Low concentrations of LPS markedly decreased the apoptosis rate of both SMMC-7721 and HepG2 cells compared to control cells; 200 μM and 100μM of castanol B (1) significantly promoted apoptosis compared to LPS-induced cells. Additionally, castanol B (1) significantlyincreased the apoptosis rate of both cells in the absence of LPS. These results indicated that the antiproliferative effect of castanol B(1) might be explained by its proapoptotic characteristic. However, the further mechanism against proliferation should be studied.

### 2.4. Effect of Castanol B (1) on LPS-Induced TNF-α and IL-6 Secretion in Hepatoma Cells

**Figure 6:**
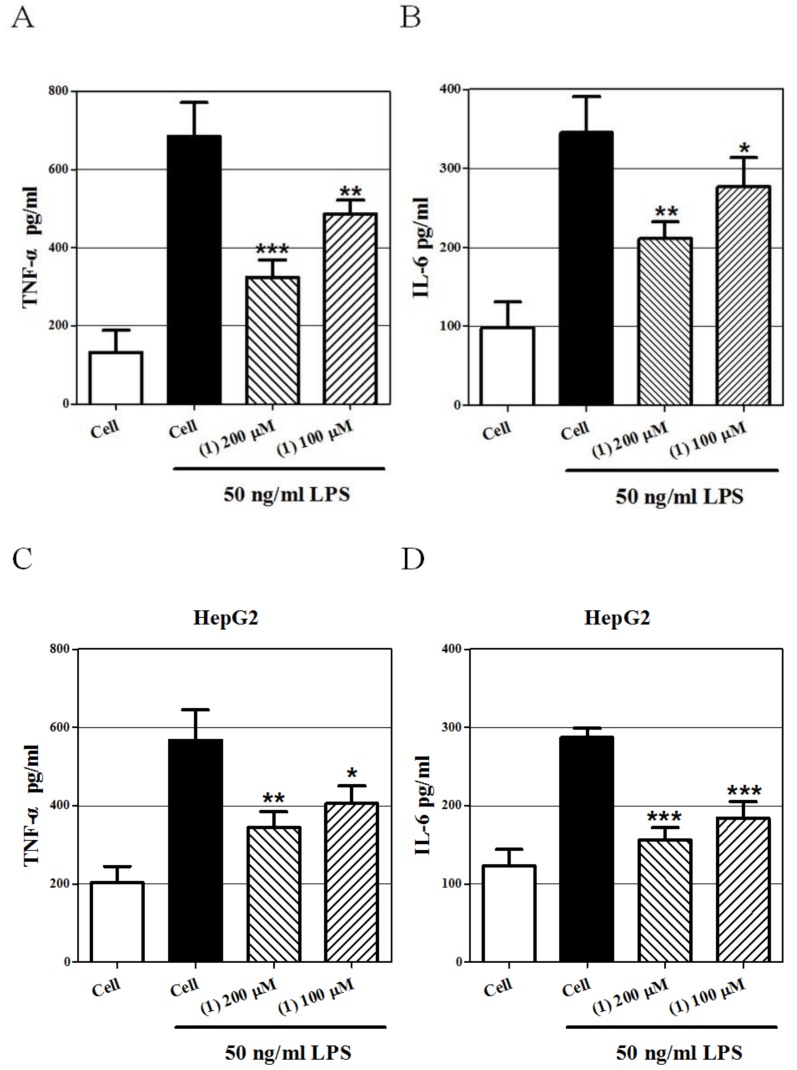
Effect of castanol B (1) on cytokines secretionon lipopolysaccharide(LPS)-induced hepatoma cells. TNF-α (**A**) and IL-6 (**B**) expression in SMMC-7721 cells was inhibited by castanol B (1)**.** TNF-α (**C**) and IL-6 (**D**) expression in HepG2 cells was inhibited by castanol B (1)**.** (* denotes *p* < 0.05; ** denotes *p* < 0.01; *** denotes *p* < 0.001, vs. LPS-induced hepatoma cells).

In order to verify the anti-inflammatory effect ofcastanol B (1)in SMMC-7721 and HepG2 cells, we measured the concentration of two key pro-inflammatory cytokines (TNF-α and IL-6) in the supernatant using ELISA. As shown in Figure 6, TNF-α and IL-6 were dramatically increased by LPS in SMMC-7721 and HepG2 cells, while their concentrations were markably decreased by 200μM and 100μM of castanol B (1) in a dose-dependent manner.

### 2.5. Effect Castanol B (1) on the TLR4–NF-κBSignal Pathway

**Figure 7:**
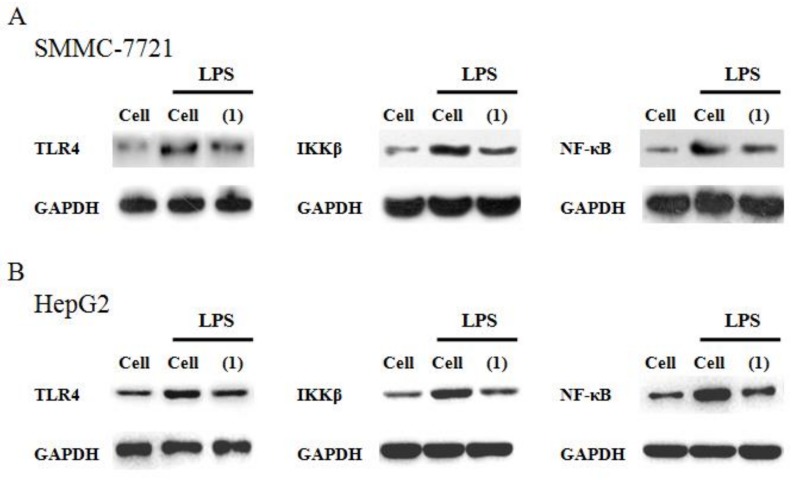
Effect of 200μM castanol B (1) on theexpression of proteins TLR4, IKKβ, and NF-κB p65 in lipopolysaccharide(LPS)-induced SMMC-7721 cells (**A**) and HepG2 cells (**B**).

In Figure 7, the expression levels of TLR4 and its downregulated proteins in the NF-κB pathway were detected by Western blot analysis. As compared to the control cells, LPS induced significant augmentation of TLR4, IKKβ, and NF-κB p65 indicating that LPS-induced TLR4 could activate the NF-κB signal pathway. However, the 200μM castanol B (1) treatment significantly prevented the activation of NF-κB p65, IKKβ, and TLR4.

## 3. Discussion

In this present study, seven compounds were purified and identified from the water-soluble extract of CMS. Castanol B (1) was a new structure compound and 7,8-dihydroxy-2H-chromen-3-one (2) was firstly reported in the physicochemical data. The parent nucleus of castanol B (1) is a biphenyl skeleton and the same as DDB, a hepatoprotective agent commonly used in treating viral hepatitis in many Asian countries and treating hepatitis B in China [9,10]. It has been also verified the inhibitory effect of DDB on invasion of human hepatoma cell with high metastasis potential [11], and the ability to reverse multidrug resistant cancer cells, such as breast carcinoma cellsin vitro [10]. In addition, castanol B (1), as one of the polyphenols, perhaps exhibits the anti-inflammatory properties of polyphenol, such as catechin and epigallocatechin. It has been considered that chronic inflammation is one of the most essential factors leading to tumorigenesis and metastatic progression of hepatocellular carcinoma [12,13]; DDB presents the properties of both anticancer and anti-inflammation, so we attempted to investigate the possible anticancer effect and mechanism of castanol B (1) against LPS-stimulated inflammatory hepatoma cells according to the link of inflammation to cancer.

LPS is a surface component of gramnegative bacteria and is known to be a strong stimulator of inflammatory response. Several studies verify that exposure of hepatoma cells to low concentration LPS enhances cell proliferation and survival, as well as induces progression of epithelial mesenchymal transition [14,15,16]. Because LPS induces both inflammatory response and proliferation on human hepatoma cells [17,18,19], it has been a representative cell model of inflammation-related hepatocellular carcinoma using LPS as stimulator. In this present study, we observed 50 ng/mL LPS induced proliferation of SMMC-7721 cells and HepG2 cells, while castanol B (1) inhibits the proliferation, which inhibitory effect might correlate its proapoptotic effect. Additionally, we found that castanol B (1) can promote the apoptosis of hepatoma cells in the absence of LPS.Because of its anti-inflammatory effect, it is proved that castanol B (1) inhibits the secretion of pro-inflammatory cytokines. Because castanol B (1) inhibits cytokines secretion and cell proliferation both in a dose-dependent manner, it speculates the correlation between anti-inflammation and anticancer by castanol B (1), and the anticancer mechanism might come from anti-inflammatory signal pathway.

LPS binds to Toll-like receptor 4 (TLR4) to activate cells in order to produce and release a large number of inflammatory cytokines, such as TNF-α and IL-6, then to trigger the inflammatory responses [20]. Activated TLR4 transduces the signal cascade to motive the transcriptional function of nuclear factor-κB (NF-κB), which is considered as the main “switch” in secreting pro-inflammatory cytokines and regulating a variety of cytokines [21]. In physical status, NF-κBbinds to its inhibitory protein, IκB, to form a complex, which is stabilized in the cytoplasm and cannot function as a transcription factor. In pathological status, IκB is phosphorylated by IKK kinase and releases NF-κB. Phosphorylated IκB is subsequently ubiquitinated and degraded by proteasome [22]. The dissociative NF-κB travels into cell nucleus, binds promoter sequences, and activates transcription of various genes leading to the transcriptional expression of downstream inflammatory molecules [23]. Thus, the TLR4–NF-κB pathway might be an important signal pathway, and might participate in LPS-induced cytokine increase and cell proliferation in hepatoma cells. In this study, castanol B (1) treatment decreases theprotein levels of three key signaling molecules in the LPS-induced TLR4–NF-κB pathway—TLR4, IKKβ, and NF-κB—suggesting the anti-inflammtory mechanism of castanol B (1). Altogether, it is verified the anticancer effect of castanol B (1) is correlated with its anti-inflammtory mechanism.

Furthermore, because castanol B (1) works at a high concentration, such as 100μM, and has the dual roles ofanticancer and anti-inflammation, its synergistic effect in combination with other agents or the ability to increase the sensitivity of other drugs should be explored.

## 4. Materials and Methods

### 4.1. General

Optical rotation was evaluated by a Bellingham and Stanley Ltd. 341 polarimeter (Bellingham and Stanley Ltd., Beaconsfield, UK). IR spectra wereobtained on a thermos Nicolet iS10 fourier transform infrared spectrometer with KBr pellets (Nicolet, Waltham, MA, USA). The UV spectrum was assessedusing a Mettle Toledo UV5 spectrometer (Mettle Toledo, Zurich, Switzerland). HRESIMS was measured with a Thermo Scientific™ 253 Uitra Q-TOF MS (Thermo Scientific™, Waltham, MA, USA). NMR spectra wereperformed by a BRUKER AVANCE III-600 spectrometer (Bruker, Germany) using TMS as an internal standard. Preparative HPLC was run on Agilent Aupos Auto Purification System comprised ofan Agilent 2545 binary gradient module, a 2767 sample manager and a 2489 UV/visible detector. Data was collected using a 1260 workstation (Agilent, Santa Clara, CA, USA). A preparative C18 column (250 × 20 mm, i.d., 10 μm) was employed in HPLC separation.

### 4.2. Plant Material

The CMS was produced in Hubei Province, China, in July 2016, and accredited by Dr. Bao-Kang Huang (Second Military Medical University, China). A voucher specimen (Zheng 5590) has been archived in the Herbarium of the Shanghai University of Traditional Chinese Medicine.

### 4.3. Water-Soluble Extract of CMS and Compound Isolation

Dried and powdered CMS was extracted twice with 10-fold weight of 40% ethanol for 2 h, and the pH value was adjusted at 3–4 using HCl. The solution was filtrated and its residue was redissolved in water. The solution was evaporated to yield sample powder. Then the sample powder (3 kg) was extracted twice with methanol (3 × 10 L) at room temperature for 15 min each. After filtration, the extracts were evaporated and dried using a rotary evaporator. Then 500 mL water was used for resolvation and was subsequently diluted 5 times. In order to eliminate baseline drift, the water solution was ultrafiltrated by a semipermeable membrane (5000 Da cutoff), then the filtrate was concentrated to 3.6 L, and the crude extract was dried and weighed 68.04 g. Finally, the crude extract was separated roughly into two parts by using self-made solid phase extraction column packed with C_18_ HC. The strong polar part and the weak polar part (21.54 g) were obtained by eluting with water (280 mL) and methanol (280 mL), respectively. The weak polar part was used for further chromatographic separation.

Preparative C_18_ ME HPLC (MeoH/H_2_O, 20%–72%, 40 min) was used to separate 12 fractions (Frs.1–12) from the weak polar part. Fr.2 was further separated by preparative C_18_ ME column (ACN/H_2_O, 5–15%, 20min) to afford two sub-fractions (Frs.2a–2b). Fr.2a was subjected to preparative C18 YE HPLC (ACN:H_2_0, 10:90) to afford compound 2 (32mg). Fr.2b was subjected to preparative C_18_ ME HPLC (ACN/H_2_O, 10–30%, 20 min) to give compounds 4 (28 mg). Fr.5 was separated by preparative C_18_ ME HPLC (ACN/H_2_O, 10–50%, 20 min) to afford compouds 6 (10 mg) and 7 (9 mg). Fr.6 was further separated by semipreparative C_18_ ME HPLC (ACN:H_2_O, 15:85) to give compounds 1 (20 mg) and 5 (29 mg). Fr.8 was subjected to preparative C18 ME HPLC (ACN/H_2_O, 20–35%, 20 min) to yield compound 3 (23 mg).

### 4.4. Castanol B (1)

Pale white and amorphous powder; ${\left[\alpha \right]}_{22}^{D}$ + 1000 (*c* = 0.001, M_e_OH); IR (KBr) *ν* (cm^−1^): 3289, 1758, 1614, 1585, 1486; UV λ max (MeOH) nm (log *δ*): 210 (3.52), 247 (4.09), 255 (3.12); HR-ESI-MS *m/z* 245.0462 (calculated 246.0533, for [M-H]^-^); ^1^H NMR (600 MHz, MeOD, δppm): 6.83(*d*, 1H, *J* =12.0 Hz, 5’-H ), δ_H_ 6.95 (*dd*, 1H, *J* = 12.0, 6.0 Hz, 6’-H), δ_H_ 7.05 (*d*, 1H, *J* = 6.0 Hz, 2’-H ), δ7.15 (*s*, 1H, 6-H), 7.33 (*s*, 1H, 4-H), 7.66 (*s*, 1H, 2-H); ^13^C NMR data: Table 1.

### 4.5. 7,8,dihydroxy-2H- chromen-3-one (2)

Pale yellow and amorphous powder; ${\left[\alpha \right]}_{22}^{D}$ + 0.667 (*c* =0.001, M_e_OH); IR (KBr) *ν* (cm^−1^): 3320, 1762, 1615,1585, 1480; UV λ max (MeOH) nm (log *δ*): 227 (3.41), 255 (4.32); HR-ESI-MS *m/z* 179.0467 (calculated 180.0426, for [M-H]^-^); ^1^H NMR (600 MHz, MeOD, δppm): 2.75, 3.42 (*m*, 2H, 4-H), 4.40 (*m*, 2H, 2-H), 6.84 (*s*, 1H, 6-H), 7.07 (*s*, 1H, 10-H); ^13^C NMR data: Table 1.

### 4.6. MTT Assay

SMMC-7721 and HepG2 cells were purchased from Fuyang biotek company (Shanghai, China). The cells were trypsinized and seeded in 96-well plates at 2000/well. After overnight culture and removal of the medium cells were cultured in serum-free medium for another 24 h. Then medium containing 1% FBS, 50 ng/mL LPS (Sigma, MA, China) and various concentrations of compounds were used to treat the cells. After 48 h treatment, cell growth was determined by cell medium containing 0.5 mg/mL MTT in each well. After incubation for 2 h, the optical density at 490 nm absorbance values was read.

### 4.7. Cell Apoptosis Assay

The hepatoma cells were trypsinized and seeded in 6-well plates at 1 × 10^5^/well, then, after overnight incubation, the medium was replaced and contained 50 ng/mL LPS and castanol B (**1**) (200 μM and 100 μM). After 48 h treatment, Annexin V-FITC/PI apoptosis kit (Beyotime, Shanghai, China) was used to determine the cell apoptosis according to the manufacturer’s protocol. Briefly, cells were collected by trypsinization and washing, then resuspended in a buffer containing Annexin V-FITC and PI at manufacturer’s specified concentrations for 15 min in the dark. Then the apoptotic cells were measured using a FACS Calibur flow cytometer (BD Biosciences, New York, NJ, USA).

### 4.8. Cytokine Assays

The concentrations of TNF-α and IL-6 (R&D system, Saint Paul, MN, USA.) in supernatants were measured using ELISA kits. The minimum detectable concentrations of both kits were 2 pg/mL.

### 4.9. Western Blotting

After 48 h of LPS stimulation and 200 μM castanol B (1) treatment, the hepatoma cells were lysed by lysis buffer and measured the total protein content by Coomassie brilliant blue assay. The proteins binding with SDS were electrophoresed on 10% SDS-PAGE and transferred to PVDF membranes. Primary antibodies were used to immune-blot the membranes overnight at 4 °C, followed by 2 h immune-blotting with secondary antibody (Boster, Wuhan, China) at room temperature. Then the bands were visualized using ECL method (Boster, Wuhan, China). Primary antibodies of TLR4, IKKβ, and NF-κB p65 were purchased from Cell Signaling Technology (Saint Paul, MN, USA).

## 5. Statistical Analysis

We used the Student’s *t*-tests to compare the differences between groups. SPSS program (v.13.0, SPSS, USA) was used to perform the statistical analysis. Results were presented as mean ± SD with *p*-value less than 0.05 as statistical significance.

## 6. Conclusions

The present study demonstrates seven phenolic compounds structures separated from water-soluble extracts of shells of *Castanea mollissima*, in which castanol B (1) is a new phenol derivative which reduces inflammation, inhibits cell proliferation, and increases cell apoptosis of human hepatoma cells via inhibiting TLR4–NF-κB pathway. This study enriches a new biphenyl derivative with physicochemical and biological activities. Furthermore, it provides new possibilities for the recycling of shells of *Castanea mollissima* (a waste from chestnut processing).

## Reference

## Figures and Tables

**Table 1:** ^13^C NMR data of compounds**1–2 **(125 MHz; in DMSO) (*δ *ppm).

No.	1	2
1	132.2	
2	118.6	73.6
3	116.9	206.7
4	113.9	34.8
5	157.5	125.8
6	116.9	111.2
7	169.9	154.6
8		148.0
9		108.2
10		148.
1’	145.1	
2’	113.5	
3’	145.2	
4’	142.6	
5’	115.3	
6’	118.0	
